# Whole Genome Sequencing of *Thermus oshimai* JL-2 and *Thermus thermophilus* JL-18, Incomplete Denitrifiers from the United States Great Basin

**DOI:** 10.1128/genomeA.00106-12

**Published:** 2013-01-24

**Authors:** Senthil K. Murugapiran, Marcel Huntemann, Chia-Lin Wei, James Han, John C. Detter, Cliff S. Han, Tracy H. Erkkila, Hazuki Teshima, Amy Chen, Nikos Kyrpides, Konstantinos Mavrommatis, Victor Markowitz, Ernest Szeto, Natalia Ivanova, Ioanna Pagani, Jenny Lam, Austin I. McDonald, Jeremy A. Dodsworth, Amrita Pati, Lynne Goodwin, Lin Peters, Sam Pitluck, Tanja Woyke, Brian P. Hedlund

**Affiliations:** a School of Life Sciences, University of Nevada Las Vegas, Las Vegas, Nevada, USA; b Los Alamos National Laboratory, Los Alamos, New Mexico, USA; c Department of Energy, Joint Genome Institute, Walnut Creek, California, USA

## Abstract

The strains *Thermus oshimai* JL-2 and *Thermus thermophilus* JL-18 each have a circular chromosome, 2.07 Mb and 1.9 Mb in size, respectively, and each has two plasmids ranging from 0.27 Mb to 57.2 kb. The megaplasmid of each strain contains a gene cluster for the reduction of nitrate to nitrous oxide, consistent with their incomplete denitrification phenotypes.

## GENOME ANNOUNCEMENT

The genus *Thermus* comprises >15 species of thermophilic bacteria, including the biotechnologically exalted *Thermus aquaticus* and the genetically tractable *Thermus thermophilus*. We reported previously the isolation of a large number of *Thermus* strains from the Great Boiling Spring system in the U.S. Great Basin, typified by *Thermus oshimai* JL-2 and *Thermus thermophilus* JL-18, and described their roles in incomplete denitrification *in situ* (1, 2).

The genomes of *T. oshimai* JL-2 and *T. thermophilus* JL-18 were sequenced using 454-GS-FLX Titanium and Illumina GA II (2 × 75 bp) methodologies, were assembled using Newbler version 2.3 (prerelease), and were annotated using Prodigal version 1.4, Gene Prediction Improvement Pipeline (GenePRIMP) at the Joint Genome Institute (JGI) in Walnut Creek, CA. The data are available at the Joint Genome Institute (JGI) Integrated Microbial Genomes (IMG) website (3). *T. oshimai* JL-2 contains a 2.07-Mb circular chromosome carrying 2,205 predicted genes; a circular megaplasmid, pTHEOS01 (0.27 Mb, 268 predicted genes); and a smaller circular plasmid, pTHEOS02 (57.2 kb, 75 predicted genes). *T. thermophilus* JL-18 has a 1.9-Mb circular chromosome carrying 2,057 predicted genes; a circular megaplasmid, pTTJL1801 (0.26 Mb, 279 predicted genes); and a smaller circular plasmid, pTTJL1802 (0.14 Mb, 172 predicted genes).

The chromosome of *T. thermophilus* JL-18 was similar to those of *T. thermophilus* HB8 and *T. thermophilus* HB27, apart from a few chromosomal rearrangements (4). However, extensive rearrangements were observed in the *T. thermophilus* JL-18 megaplasmid pTTJL1801 when compared to the *T. thermophilus* HB8 and HB27 megaplasmids; this is consistent with the elevated plasticity reported previously for *Thermus* megaplasmids (5). The chromosome and megaplasmid of *T. oshimai* JL-2 pTHEOS01 exhibit little or no synteny with any of the complete *Thermus* genomes.

The megaplasmids of both species include a complete nitrate reductase operon (*narGHJIK*). *T. oshimai* JL-2 possesses two nitrate/nitrite transporters (*narK1* and *narK2*), which is similar to *T. thermophilus* HB8 (6), whereas *T. thermophilus* JL-18 possesses a single copy of *narK*. Genes encoding nitrite reductase (*nirS* and *nirK* in *T. oshimai* JL-2 and *nirS* in *T. thermophilus* JL-18) and nitric oxide reductase (*norB* and *norC*) were also identified in close proximity to the *narGHIJK* operon in the megaplasmids of both *T. thermophilus* JL-18 and *T. oshimai* JL-2. However, nitrous oxide reductase (*nos*) genes, which are needed for the conversion of nitrous oxide to dinitrogen, are absent, concurrent with the incomplete denitrification phenotype of these strains and the high flux of nitrous oxide reported at Great Boiling Spring (2). Both megaplasmids also possess genes encoding a DNA repair system that is proposed to impart thermophily in *T. thermophilus* HB8 and HB27 (5). A *sox* gene cluster that includes a sulfite dehydrogenase gene (*soxCD*) essential for the chemotrophic growth of *Paracoccus pantotrophus* (7) was identified in *T. oshimai* JL-2 and *T. thermophilus* JL-18 chromosomes, suggesting that these organisms can carry out sulfur oxidation.

### Nucleotide sequence accession numbers.

The genome sequences from this study are available from GenBank under the following accession no: CP003249.1 (*T. oshimai* JL-2 chromosome), CP003250.1 (*T. oshimai* JL-2 megaplasmid pTHEOS01), CP003251.1 (*T. oshimai* JL-2 plasmid pTHEOS02), CP003252.1 (*T. thermophilus* JL-18 chromosome), CP003253.1 (*T. thermophilus* JL-18 plasmid pTTJL1801), and CP003254.1 (*T. thermophilus* JL-18 plasmid pTTJL1802).
